# Characterization of *MTAP* Gene Expression in Breast Cancer Patients and Cell Lines

**DOI:** 10.1371/journal.pone.0145647

**Published:** 2016-01-11

**Authors:** Sarah Franco Vieira de Oliveira, Monica Ganzinelli, Rosaria Chilà, Leandro Serino, Marcos Euzébio Maciel, Cícero de Andrade Urban, Rubens Silveira de Lima, Iglenir João Cavalli, Daniele Generali, Massimo Broggini, Giovanna Damia, Enilze Maria de Souza Fonseca Ribeiro

**Affiliations:** 1 Department of Genetics, Federal University of Paraná, Curitiba, Paraná, Brazil; 2 Laboratory of Molecular Pharmacology, Department of Oncology, Istituto di Ricerche Farmacologiche ‘‘Mario Negri”, Milan, Lombardia, Italy; 3 Department of Mastology, Breast Unit, Hospital Nossa Senhora das Graças, Curitiba, Paraná, Brazil; 4 Laboratorio di Oncologia Molecolare Senologica, U. O. Multidisciplinare di Patologia Mammaria, A. O. Istituti Ospitalieri di Cremona, Cremona, Lombardia, Italy; Cleveland Clinic Lerner Research Institute, UNITED STATES

## Abstract

*MTAP* is a ubiquitously expressed gene important for adenine and methionine salvage. The gene is located at 9p21, a chromosome region often deleted in breast carcinomas, similar to *CDKN2A*, a recognized tumor suppressor gene. Several research groups have shown that *MTAP* acts as a tumor suppressor, and some therapeutic approaches were proposed based on a tumors´ *MTAP* status. We analyzed *MTAP* and *CDKN2A* gene (RT-qPCR) and protein (*western-blotting*) expression in seven breast cancer cell lines and evaluated their promoter methylation patterns to better characterize the contribution of these genes to breast cancer. Cytotoxicity assays with inhibitors of *de novo* adenine synthesis (5-FU, AZA and MTX) after *MTAP* gene knockdown showed an increased sensitivity, mainly to 5-FU. *MTAP* expression was also evaluated in two groups of samples from breast cancer patients, fresh tumors and paired normal breast tissue, and from formalin-fixed paraffin embedded (FFPE) core breast cancer samples diagnosed as Luminal-A tumors and triple negative breast tumors (TNBC). The difference of *MTAP* expression between fresh tumors and normal tissues was not statistically significant. However, *MTAP* expression was significantly higher in Luminal-A breast tumors than in TNBC, suggesting the lack of expression in more aggressive breast tumors and the possibility of using the new approaches based on *MTAP* status in TNBC.

## Introduction

Breast cancer is the most common cancer among women worldwide [1–2]. One of the alterations involved in the development and progression of the disease is the loss of expression of tumor suppressor genes [3].

The methylthioadenosine phosphorylase (*MTAP*) gene is located at 9p21 and is flanked by the tumor suppressor *miR-31* and the cyclin-dependent kinase inhibitor 2A (*CDKN2A*) gene [4], which are approximately 100 kb away [5–9]. MTAP acts in the polyamine biosynthesis pathway and is important to the salvage of both adenine and methionine [3]. *MTAP* is ubiquitously expressed in all normal tissues but frequently lost in tumors mainly due to a co-deletion with *CDKN2A*. In normal cells, MTAP cleaves the 5’-deoxy-5’-methylthioadenosine (MTA) substrate generated during the biosynthesis of polyamines, generating adenine and 5-methylthioribose-1-phosphate (MTR-1-P). Adenine is converted to adenosine monophosphate (AMP), and MTR-1-P is converted to methionine. Cells lacking *MTAP* are unable to salvage AMP or methionine and are more sensitive to inhibitors of *de novo* AMP synthesis or to methionine starvation than normal cells [10–11]. Because MTAP is expressed in all normal tissues and is usually lost in tumors, Kadariya et al. [12] suggested using *MTAP* deficiency to selectively target tumor cells that are *MTAP*-negative. A promising therapeutic approach to cancer was proposed in 2009 by Lubin and Lubin [13], based on the addition of MTA to the treatment of *MTAP*-negative tumors with toxic purine analogs, like 5-fluorouracil (5-FU). Normal cells are protected from the toxic effects of purine analogs by the AMP produced from MTA. However, *MTAP*-negative tumor cells are not able to produce AMP from the added MTA, so the purine analogs are metabolized and exert their toxic effects [14].

We have previously reported a 90% frequency of concordant loss of heterozygosity (LOH) for intragenic microsatellite markers for *CDKN2A* (D9S1748) and *MTAP* (D9S1749) [15]. These data indicated that in breast cancer cells, the co-deletion might play an important role, as described in other types of tumors (3–9). The aim of the present study was to characterize *MTAP* expression in breast cancer patients and cell lines and examine the relationship between *MTAP* expression and chemo-sensitivity to inhibitors of AMP synthesis.

## Materials and Methods

### Ethics Statement

This research was approved by the “Comissão Nacional de Ética em Pesquisa (CONEP)”, from the Health Division of Brazilian Government, number 251/2003. The Ethical Committee from the Istituto Ospitalieri di Cremona (Italy) approved the use of formalin-fixed paraffin embedded (FFPE) samples. Written informed consent was obtained from all patients. All of the samples were anonymized by a pathologist staff member, and none of the researchers conducting the analysis had access to the clinico-pathological data.

### Fresh tumors

Forty-six fresh primary breast tumors were obtained from 45 patients between 2009 and 2011 at the *Hospital Nossa Senhora das Graças* (Curitiba, Brazil). Non-compromised tissues of the contralateral breast were obtained from ten patients who underwent simultaneous breast symmetrization. Histological analysis confirmed the normality of these samples. Tumor and normal samples were conserved in an RNA stabilization solution (RNAlater^®^, Applied Biosystems, USA) immediately after surgery, and stored at 4°C until RNA isolation. Clinico-pathological information of the patients are summarized in Table 1. Patients had received neither chemotherapy nor radiation prior to surgery.

**Table 1:** Clinico-pathological information of primary breast tumors.

Subtypes	Number of patients	%
IDC	35	76.09
ILC	3	6.52
Mixed	4	8.70
Others^a^	4	8.70
*Intrinsic Subtypes*		
Luminal-A	23	54.80
Luminal-B	14	33.30
HER2 +	2	4.80
TNBC	3	7.14
Not-informed	4	
*Lymph node metastasis*		
Present	21	48.84
Absent	22	51.16
Not-informed	3	
*Tumor grade*		
I	6	13.64
II	28	63.64
III	10	22.72
Not-informed	2	
*Tumor size*		
≤ 20 mm	20	45.45
> 20 mm	24	54.55
Not-informed	02	
*Estrogen receptor (ER)*		
Positive	37	84.09
Negative	7	15.91
Not-informed	2	
*Progesterone receptor (PR)*		
Positive	38	86.36
Negative	6	13.64
Not-informed	2	
*HER2 amplification*		
Positive	9	21.43
Negative	33	78.57
Not-informed	4	

IDC, invasive ductal carcinoma; ILC, invasive lobular carcinoma; TNBC, triple negative breast cancer; mm, millimeters.

^a^Mucinous carcinoma, tubular-lobular carcinoma, pleomorphic carcinoma.

### Formalin fixed-paraffin embedded (FFPE) samples

For *MTAP* gene expression in FFPE samples, a second group of 81 TNBC and 60 Luminal-A breast tumors were retrospectively collected from patients who came to the medical observation facility at the Breast Care Unit, A.O. *Istituti Ospitalieri di Cremona*, Italy. We did not have access to the clinico-pathological data of these patients. The histological classification of Luminal A, Luminal B, HER2 positive and TNBC tumors was based on St. Gallen guidelines [16], which define breast tumors according to the immunohistochemical staining of hormonal receptors (ER and PR), HER2 expression, the Ki-67 marker and histological grade [17–18].

### Cell lines and cell culture

Breast cancer cell lines MDA-MB-231, MDA-MB-435S, MDA-MB-468, MCF7, SK-BR-3, T47-D and ZR-75-1 were obtained from the American Type Culture collection (ATCC^®^). Cell line authentication was performed within the last 6 months. These cell lines were grown in RPMI-1640 medium supplemented with 1% L-glutamine (Biowest, French) and 10% fetal bovine serum (FBS; Sigma, EUA) at 37°C and 5% of CO_2_. Cell growth was evaluated in control not transfected, in scramble transfected and in esiRNA *MTAP* transfected MDA-MB-435 cells at different time points after transfection using MTS (3-(4,5-dimethylthiazol-2-yl)-5-(3-carboxymethoxyphenyl)-2-(4-sulfophenyl)-2H-tetrazolium) assay, following the manufacturer’s description (Promega).

### Reverse transcribed quantitative PCR (RT-qPCR) and Methylation specific PCR (MS-PCR)

RNA from fresh tumors and normal samples were isolated using an RNAeasy^*®*^ Kit (Qiagen, Germany). RNA from FFPE samples was isolated using High Pure RNA Paraffin Kit (Roche, USA). RNA from cell lines was isolated using SV-Total RNA isolation system (Promega, USA). All mRNAs were reverse-transcribed using the High Capacity cDNA Archive Kit (Applied Biosystems, USA). The integrity of all the RNA preparations was checked on a 1% agarose gel and RNA concentrations were measured with a NanoDrop^™^ 1000 spectrophotometer (Thermo Scientific, USA). Optimal primer pairs (S1 Table) were chosen, spanning splice junctions, using PRIMER-3 (http://frodo.wi.mit.edu/cgi-bin/primer3/primer3_www.cgi) and *Oligo Analyzer* software (http://www.idtdna.com/analyzer/applications/oligoanalyzer/). The specificity was verified by detecting single-band amplicons of the PCR products. For the fresh samples, reactions were performed with 15 ng of cDNA template, 2 pmol of forward and reverse primers and 5 μl of SYBR Green PCR Master Mix (Applied Biosystems), and a dissociation curve was evaluated. Standard curves for each gene were included for efficiency reaction analysis, which is necessary to quantify expression based on the comparative method (-DDCt). Samples were then normalized using the housekeeping genes Actin (*ACTB*) and β-2-microglobulin (*B2M*). These gene were chosen as housekeeping genes based on the TaqMan^®^ Human Endogenous Control Array microfluidic card (Applied Biosystems, USA). The normalized values were compared with expression in the MDA-MB-231 cell line, which is used as a calibrator sample. The data are described as fold changes in gene expression relative to the calibrator sample. Relative *MTAP* expression in fresh samples was determined on a Mastercycler ep RealPlex System (Eppendorf, Germany). Gene expression was performed using GoTaq qPCR Master Mix (Promega, USA) in an ABI Prism 7900 Sequence Detection System (Applied Biosystems, USA).

To perform MS-PCR, genomic DNA was isolated from six breast cancer cell lines (except SK-BR-3 cell line) using the Maxwell^®^ 16 Cell DNA Purification Kit (Promega, USA). Genomic DNA was modified with sodium bisulfite using the Epitect Bisulfite Kit (Qiagen, Germany) according to manufacturer specifications. MS-PCR was performed in standard conditions using GoTaq^®^ Hot Start (Promega, USA) and 2 μl of modified DNA. By targeting the CpG island sequence in the promoter region of the *MTAP* gene, specific primers recognizing methylated or non-methylated DNA were designed (S1 Table). MS-PCR products were separated on 2.5% agarose gels. The experiment was repeated twice.

### Western-blotting (WB)

Protein extracts from seven breast cancer cell lines were obtained using a lysis buffer (10 mM Tris-HCl (pH 7.4); 150 mM NaCl, 0.1% NP-40; 5 mM EDTA; 50 mM NaF) in the presence of protease inhibitors. Total cellular proteins (50 and 100 μg for CDKN2A and MTAP analysis, respectively) were separated on 12% SDS-polyacrylamide gels and electro-transferred onto nitrocellulose or polyvinylidene fluoride (PVDF) membranes (Protran, Schleicher & Schuell, Germany). Immunoblotting was carried out with rabbit anti-human p16 (CDKN2A) (C-20)-G polyclonal antibody, goat anti-MTAP polyclonal antibody (N-20) and rabbit anti-β-tubulin (H-235) polyclonal antibody (dilution 1:200, 1:100 and 1:500, respectively), purchased from Santa Cruz Biotechnology Inc. (Dallas, Texas, USA). Membranes were then reacted with secondary antibodies (dilution 1:3000 in blocking buffer, Santa Cruz Biotechnology) and developed using the ECL Kit (Amersham Biosciences, Sweden).

### esiRNA transfection

Double-strand esiRNA for *MTAP* and a non-specific scrambled siRNA were obtained commercially (Eupheria Biotech, Sigma). Fifty microliters of the *MTAP*-positive MDA-MB-435 cells were seeded in a 96-well culture vessel at 4.0 x 10^4^ cells/ml the day before transfection. Cells were transfected with esiRNA duplexes or scrambled siRNA (45 nmol each) using Lipofectamine 2000 reagents (Invitrogen) according to the vendor’s protocol. MTAP expression was determined using WB.

### Wound Healing assay

The effect of *MTAP* downregulation on the invasiveness of MDA-MB-435 cells was evaluated with a wound healing assay. Cells were seeded in 24-well culture plates at 4 x 10^4^ cells/mL and after 24 hours were transfected with scramble siRNA or *MTAP* esiRNA in duplicate, as already described. 48 hours later a wound was made along the diameter of each well and a picture was taken. Cells were allowed to proliferate and to invade the wound for the next 96 hours and pictures of the wells were taken. Using ImageJ software the wounded area uncovered by cells was obtained for each picture, the mean of the duplicates was calculated and then expressed as percentage of the initial wounded area.

### Cytotoxicity experiments

Seventy-two hours after esiRNA transfection, cells were treated for 72 hours with crescent concentrations of 5’aza-deoxycytidine (AZA), methotrexate (MTX) and 5-FU at a final concentration of 100 μM, 50 μM and 10 μM, respectively. These drugs were chosen due to their action as inhibitors of *de novo* AMP synthesis. Cell proliferation was measured in a TECAN^®^ Infinite 200 multimode microplate reader (TECAN Group Ltd., Switzerland), using the MTS assay as already described. These experiments were performed in triplicates and repeated twice.

### Statistical analysis

RT-qPCR data had a non-normal distribution and were analyzed using the Shapiro-Wilk normality test. The Mann-Whitney U test was chosen to compare clinico-pathological and gene expression data between the groups (fresh tumors and normal samples, TNBC and luminal-A tumors), and a linear regression was chosen to correlate gene expression with patient’s age and tumor size. Statistical analyses were carried out in Prism 5 version 5.04 (GraphPad Software Inc., USA). All statistical tests were two-tailed and *p values* <0.05 were considered statistically significant.

## Results

### *MTAP* expression in breast cancer samples

**Fig 1:**
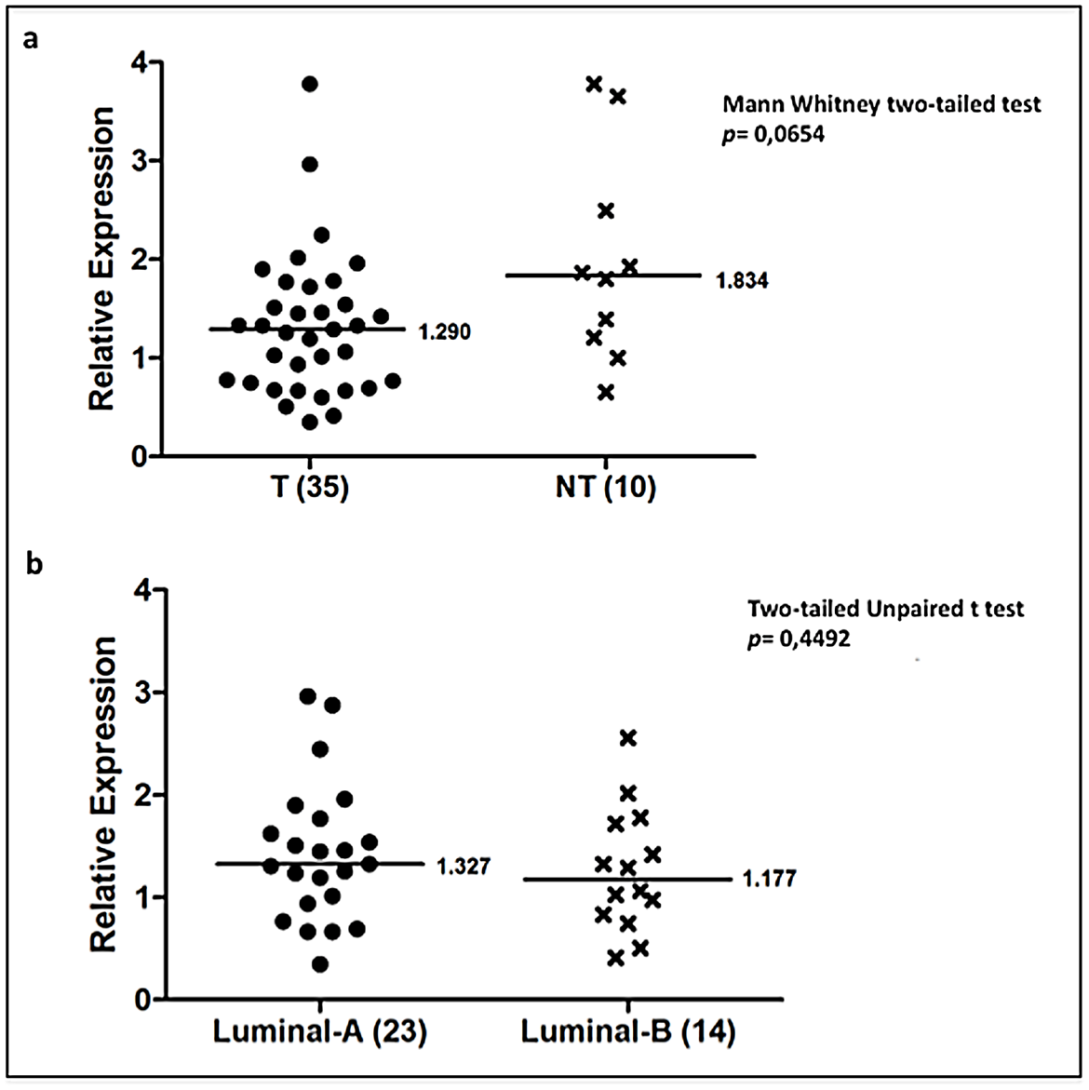
Scatter-plot graphics of *MTAP* expression in fresh primary breast tumors. (**a**) IDC and normal samples. Black line represents the median value. T, IDC tumors; NT, normal (non-tumor) samples; (number of samples). (**b**) Luminal-A and Luminal-B fresh samples.

**Fig 2:**
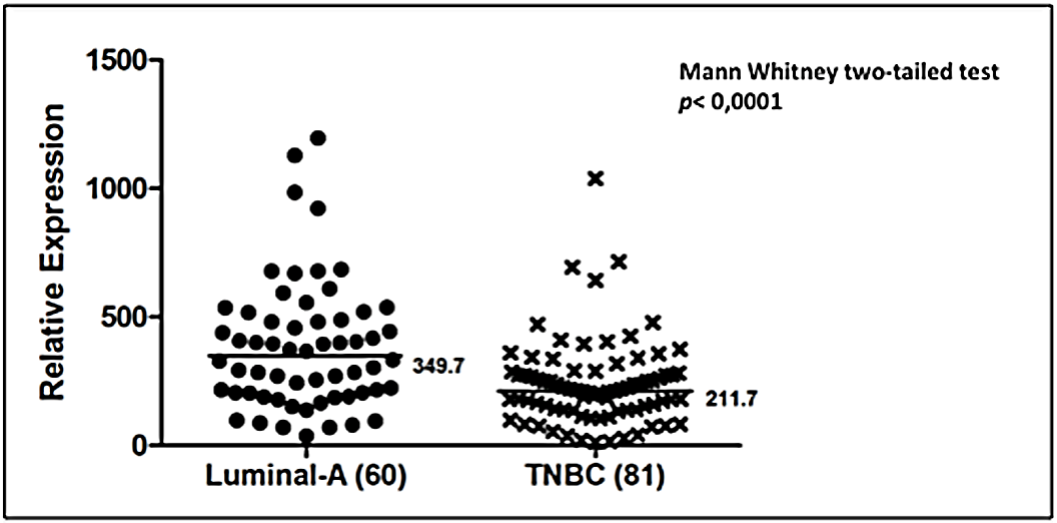
Scatter-plot graphic of *MTAP* expression in FFPE breast cancer samples. Luminal-A and TNBC. Black line represents the median value.

Relative *mRNA MTAP* expression value was 1.39 ± 0.75 and 1.98 ± 1.05 in fresh primary breast tumors and normal samples, respectively. The difference was not statistically significant (*p* value = 0.09). To evaluate a more homogeneous group, we analyzed Invasive Ductal Carcinoma (IDC) samples separately (35 patients). The relative expression value was 1.32 ± 0.72 and 1.98 ± 1.05 in IDC and normal samples, respectively and this difference was not statistically significant (*p* value = 0.065) (Fig 1a). For the IDC samples, no significant differences were found between mRNA *MTAP* expression and the clinico-pathological parameters (axillary lymph node metastasis, ER/PR/HER2 status, tumor grade, tumor size and age at diagnosis). When the different intrinsic subtypes were considered, no difference in mRNA *MTAP* level was observed between Luminal-A (n = 23) and Luminal-B (n = 14) groups (1.43 ± 0.68 and 1.26 ± 0.6, respectively, *p* value = 0.45) (Fig 1b). Expression of *MTAP* mRNA was also evaluated in FFPE breast cancer samples *MTAP* mRNA level was 1.62 times greater in Luminal-A than in TNBC, and this difference was statistically significant (*p* value< 0.0001) (Fig 2).

### *MTAP* and *CDK2A* status in cell lines

**Fig 3:**
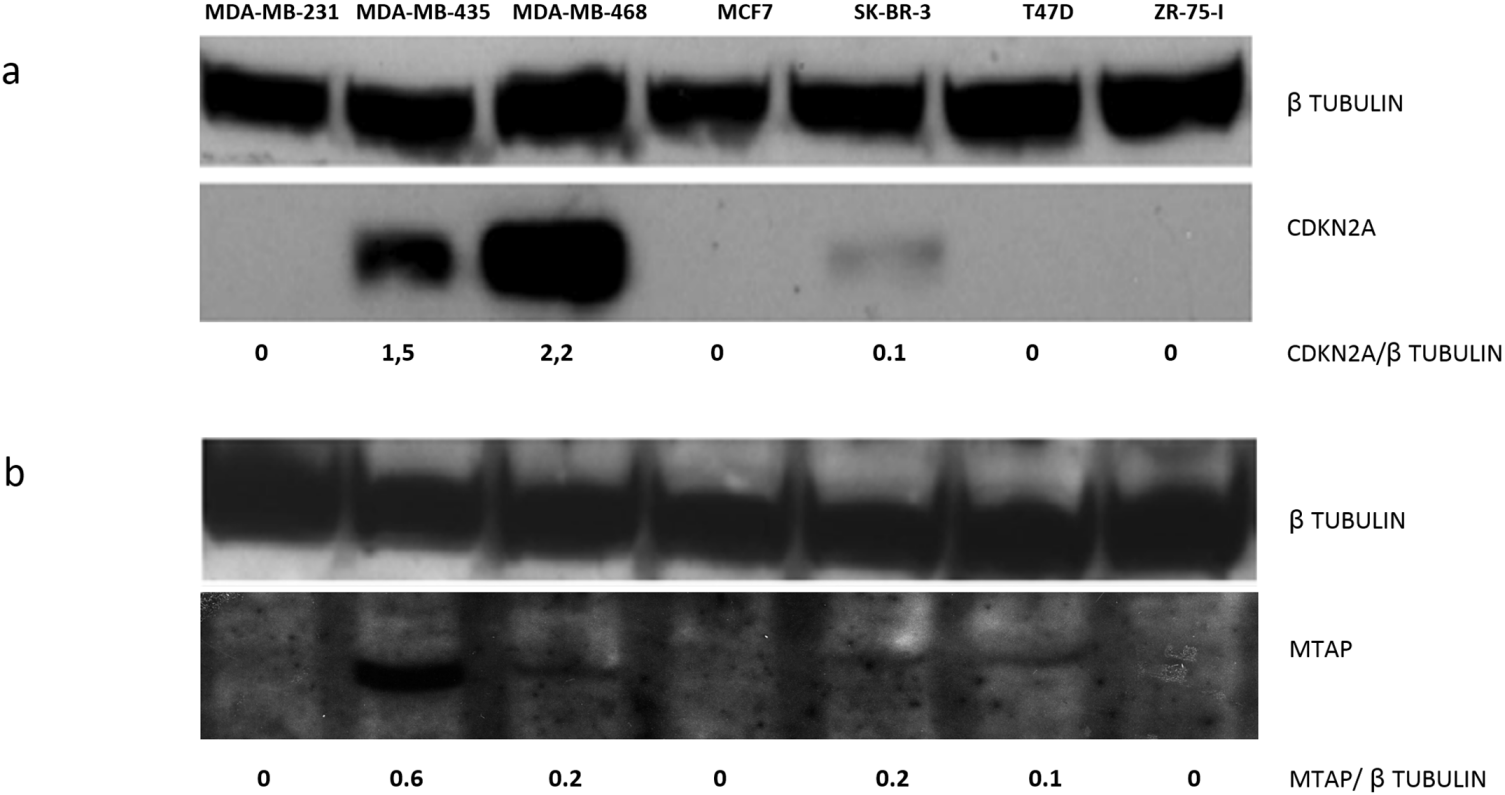
Western-blotting at breast cancer cell lines. (**a**) *CDKN2A*. (**b**) *MTAP*. β-tubulin protein; *CDKN2A* protein; *MTAP* protein. Numbers represent the ratio.

*MTAP* mRNA levels were evaluated in seven breast cancer cell lines. We also investigated the expression of the *CDKN2A* gene to determine the presence or absence of a co-deletion. *CDKN2A* and *MTAP* mRNAs were not detected in MDA-MB-231, ZR-75-I or MCF-7 cells. In the other cell lines (MDA-MB-435, MDA-MB-468, SK-BR-3 and T47-D), different expression levels of *CDKN2A* and *MTAP* were observed (Table 2).CDKN2A protein was readily detected in MDA-MB-435, MDA-MB-468 and SK-BR-3, whereas MTAP protein was clearly detected in only MDA-MB-435 and MDA-MB-468 cells and at a much lower extent in SK-BR-3 and T47-D cells (Fig 3a and 3b and Table 2). The MDA-MB-435 cell line showed the strongest *MTAP* mRNA and protein expression. MDA-MB-468 and SK-BR-3 cell lines showed intermediate mRNA expression and no protein expression, whereas the T47D cell line showed weak mRNA expression and no protein expression. Cell lines MDA-MB-231, MCF-7 and ZR-75-I did not show mRNA or protein expression. We observed an equal distribution of *MTAP* expression among cell lines in respect to their ER, PR and HER2 expression; indeed among the cell lines positive to the hormonal receptors, two (MCF-7 and ZR-75-1) were negative and T-47-D showed a weak expression. Among the hormonal receptors negative ones, two expressed *MTAP*, MDA-MB-435, MDA-MB-468, and two MDA-MB-231and SKBR3 did not. We performed MS-PCR in breast cancer cell lines to evaluate the *MTAP* promoter status. Four of the *MTAP* promoters were partially methylated (MDA-MB-435, MDA-MB 468, MCF-7 and T47-D), whereas in MDA-MB-231 and ZR-75-I cells, no methylated or unmethylated bands could be detected (S1 Fig and Table 2).

**Table 2:** mRNA, protein and methylation status of *CDKN2A* and *MTAP* in the breast cancer cell lines.

	RT-qPCR *CDKN2A*	RT-qPCR *MTAP*	WB CDKN2A	WB MTAP	MS-PCR *MTAP*
MDA-MB-231	ND	ND	ND	ND	ND
ZR-75-1	ND	ND	ND	ND	ND
MCF-7	ND	ND	ND	ND	M/U
MDA-MB-435	1.00	1.00	++	+++	M/U
MDA-MB-468	1.49	0.46	+++	++	M/U
SK-BR-3	0.57	0.42	+	+	NP
T47-D	4.46	0.19	ND	+	M/U

RT-qPCR, quantitative Real-Time PCR (relative data); ND, not detected (without expression); WB, western-blotting; MS-PCR, methylation specific PCR; +, ++, +++, level of expression; NP, not performed; M/U, methylated/unmethylated.

### esiRNA transfection and cytotoxicity experiments

**Fig 4:**
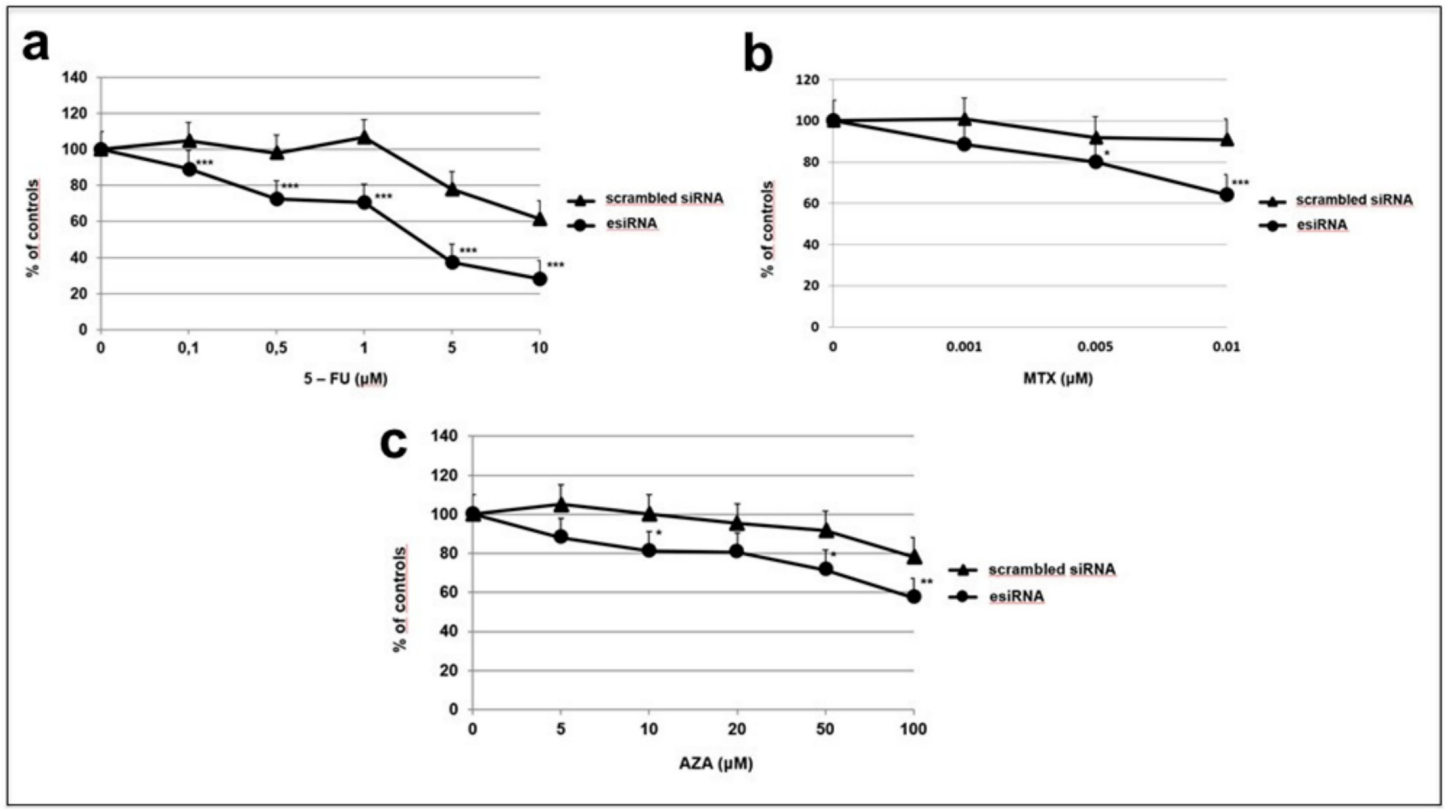
*MTAP* inactivation by esiRNA transfection and cell viability after AMP inhibitors treatment. (**a**) Inhibition of *MTAP*- cell viability by 5-FU. MTAP+ (control) and *MTAP*- cells were exposed to 5-FU concentrations ranging from 0–10 μM. Data are expressed as % of controls. (**b**) Same as (a) except MTX doses ranging from 0–0.01 μM were used instead of 5-FU. (**c**) Same as (a) except AZA doses ranging from 0–100 μM were used instead of MTX. Mean ± SD of two different experiments done in quintuplicate. *, *p*< 0.05; **, *p*< 0.005; ***, *p*< 0.0005, compared with the corresponding control.

Because MTAP levels have been shown to influence the cytotoxicity of some anticancer agents [14],the MDA-MB-435 cell line was chosen to perform knockdown of the *MTAP* gene using the esiRNA system. Transfection of esiRNA against MTAP completely knocked down MTAP protein and no differences in cell growth and cell migration, assessed by the wound healing assay were detected. S2 Fig). However, MTAP knockdown increased the cytotoxic activity of 5FU, MTX and Aza (Fig 4a–4c).

## Discussion

Curtis and colleagues [19] discovered a high frequency of *MTAP* deletions in an integrated analysis of copy number and gene expression with two sets of almost a thousand primary breast tumors. Nevertheless, there is a lack of information on *MTAP*-deficiency in primary breast cancer [4]. In a previous study [15], we found a high rate (90%) of concordant LOH between *CDKN2A* and *MTAP* genes in primary breast tumors. Here, we assessed *MTAP* mRNA expression in a sample of fresh breast tumors and normal breast tissue, and the difference was not statistically significant (Fig 1a). In addition, we did not find any correlation between *MTAP* expression and the clinico-pathological parameters, probably due the small size of our sample. Miyazaki et al. [20], in a cohort of 40 osteosarcoma samples, found a 27.5% decrease in MTAP protein expression and no correlations with the clinico-pathological parameters. Our results are similar to the findings of Alhebshi et al. [21], who reported MTAP protein expression in 20 normal human skin tissue samples and 109 cutaneous squamous cell carcinomas and found no significant correlations with the clinico-pathological parameters. The small size of our sample and contamination with normal cells after macro-dissection of the fresh tumors may be responsible for the results we obtained. We studied a second group of FFPE samples and found significantly higher expression of *MTAP* in Luminal-A tumors than of TNBC (Fig 2). Christopher et al. [7] observed that the loss or reduction of MTAP expression in breast tumor cells is involved in anchorage-independent growth. This process is important for the progression of the disease, allowing the tumor to spread and metastasize. These characteristics are commonly observed in the more aggressive cancers like TNBC. Crespo et al. [22] noted the potential relevance of *MTAP* as a tumor suppressor in glioblastomas because *MTAP* was the single homozygously deleted gene at chromosome 9p21 (from 11 genes analyzed at this region) for which they found a high correlation between copy number values and mRNA expression levels. However, Dou et al. [23] observed an inverse correlation between cellular differentiation and *MTAP* relative expression in colorectal cancer, mainly due to promoter demethylation in more malignant tumors. Tang et al. [24] demonstrated that the tumor suppressor function of *MTAP* in HT1080 fibrosarcoma cells is not the same as its known enzymatic function.

*MTAP* loss can be associated with *CDKN2A* loss [10–12, 25], and promoter hypermethylation has been described as an alternative mechanism for the loss of *MTAP* expression [26]. Here, we characterized gene expression (mRNA and protein levels) of MTAP and CDKN2A in seven breast cancer cell lines and performed a promoter methylation analysis of *MTAP* (Fig 3, Table 2 and S1 Fig). MCF-7 and MDA-MB-231 cells were already known to be *MTAP*-deficient [4]. Our results suggest that cell lines MDA-MB-231 and ZR-75-I harbor a co-deletion of *MTAP* and *CDKN2A* genes because neither gene was amplified by RT-qPCR and MS-PCR. In addition, protein expression of neither gene was detected by Western blotting. However, the methods used cannot completely exclude this possibility. In our study, MCF-7 cells show no expression of *CDKN2A* and *MTAP*; however. the *MTAP* promoter was partially methylated. Bisogna et al. [27] also described a deletion of *CDKN2A* in this cell line but not a deletion of *CDKN2B* or *INK4A* genes, which are closely located on the chromosome. Perhaps this is not a case of co-deletion, and *MTAP* is not expressed in MCF-7 cells due to DNA methylation. T47-D cells show strong *CDKN2A* mRNA expression but no evidence of protein expression by Western blotting. Bisogna et al. [27] observed DNA methylation of the *CDKN2A* promoter in this cell line, which could explain the absence of protein in our study. However, the presence of mRNA suggests post-transcriptional regulation, for example, via RNA interference. Kim et al. [3] analyzed a set of gastric cancer cell lines and found a correlation between mRNA down-regulation and homozygous deletion of *MTAP* and *CKN2A* because 8 of 10 cell lines expressed both genes. However, the proteins were absent in two out of ten cell lines with a homozygous deletion. We observed no difference in mRNA *MTAP* expression in respect to their ER, PR and HER2 expressionamong the cell lines and these data partially constrast with the fact that in FFEE triple negative samples did express higher MTAp mRNA levels. The low number of cell lines considered can be at the basis of this discrepancy.

Hellerbrand et al. [28] showed a down-regulation of *MTAP* in 15 hepatocellular carcinoma (HCC) samples. Another study [29] demonstrated that down-regulation of *MTAP* increases MTA levels in HCC, which could be involved in HCC progression. Myiazaki et al. [20] proposed that the MTAP enzyme deficiency observed in osteosarcomas was caused by genetic and epigenetic mechanisms and that MTAP deficiency could be exploited using selective chemotherapy with inhibitors of *de novo* polyamine synthesis. Zimling, Jorgensen and Santoni-Rugiu [30], studying MTAP immunoreactivity in 99 malignant pleural mesotheliomas (MPMs), found that 65% of the tumors analyzed had a decreased reactivity to MTAP. They proposed that this decreased MTAP expression, in combination with other common markers, could be a potential diagnostic marker. As for MPMs, the decreased expression of *MTAP* in TNBC (Fig 2) could be useful as a diagnostic and therapeutic marker.

Several different approaches based on MTAP status have proposed to use inhibitors of *de novo* purine synthesis and the enzyme substrate MTA to selectively kill MTAP-negative cells [4, 11, 13, 31–33]. Our gene expression knockdown experiments support the therapeutic approach proposed by Lubin and Lubin [13], once our data show a significantly higher sensitivity of MTAP-negative cells to 5-FU (Fig 4a). Interestingly, our data show that TNBC cells express significantly less *MTAP* than the more differentiated group composed of Luminal-A breast tumors (Fig 2), which may open the possibility of this new approach to TNBC patients who lack the benefit of endocrine or targeted therapy that is largely used in Luminal and HER2 groups.

## Conclusions

This work investigated *MTAP* expression in breast cancer patients and cell lines, and examined the relationship between *MTAP* expression and chemo-sensitivity to inhibitors of AMP synthesis. *MTAP* was found significantly less expressed in TNBC than in Luminal-A breast tumors. We observed that after gene knockdown, *MTAP*-negative cells were significantly more sensitive to 5-FU, MTX and AZA. The observation that TNBC tumors have lower levels of MTAP has to be corroborated in additional studies, but the observation suggests that this class of patients could benefit from treatment with antimetabolites.

## Supporting Information

S1 Fig *MTAP* MS-PCR at breast cancer cell lines. M, methylated; U, unmethymated.
(PPTX)

S2 Fig Effect of MTAP downregulation on the proliferation and the invasiveness of MDA-MB-435 cells. A. Western-blotting of MTAP, CDKN2A and β-Tubulin proteins at 48 and 72 hours from transfection with scramble siRNA and MTAP esiRNA. B. Proliferation of cells untransfected, transfected with scramble siRNA and transfected with MTAP esiRNA expressed as value of adsorbance at the wavelenght of 490 nm at different time points. C. Invasiveness of cells untransfected, transfected with scramble siRNA and transfected with MTAP esiRNA expressed as percentage of the uncovered area versus the initial one at different times from the wound.
(PPTX)

S1 Table RT-qPCR and MS-PCR primers. Primers sequences are in the sense 5’- 3’. m, methylated; u, unmethylated.
(DOCX)
