# Supplementary figures and images for: Characterization of MTAP Gene Expression in Breast Cancer Patients and Cell Lines
Source: PLoS One. 2016 Jan 11;11(1):e0145647. doi: 10.1371/journal.pone.0145647 (PMC4709099; doi:10.1371/journal.pone.0145647)

## Slide 1
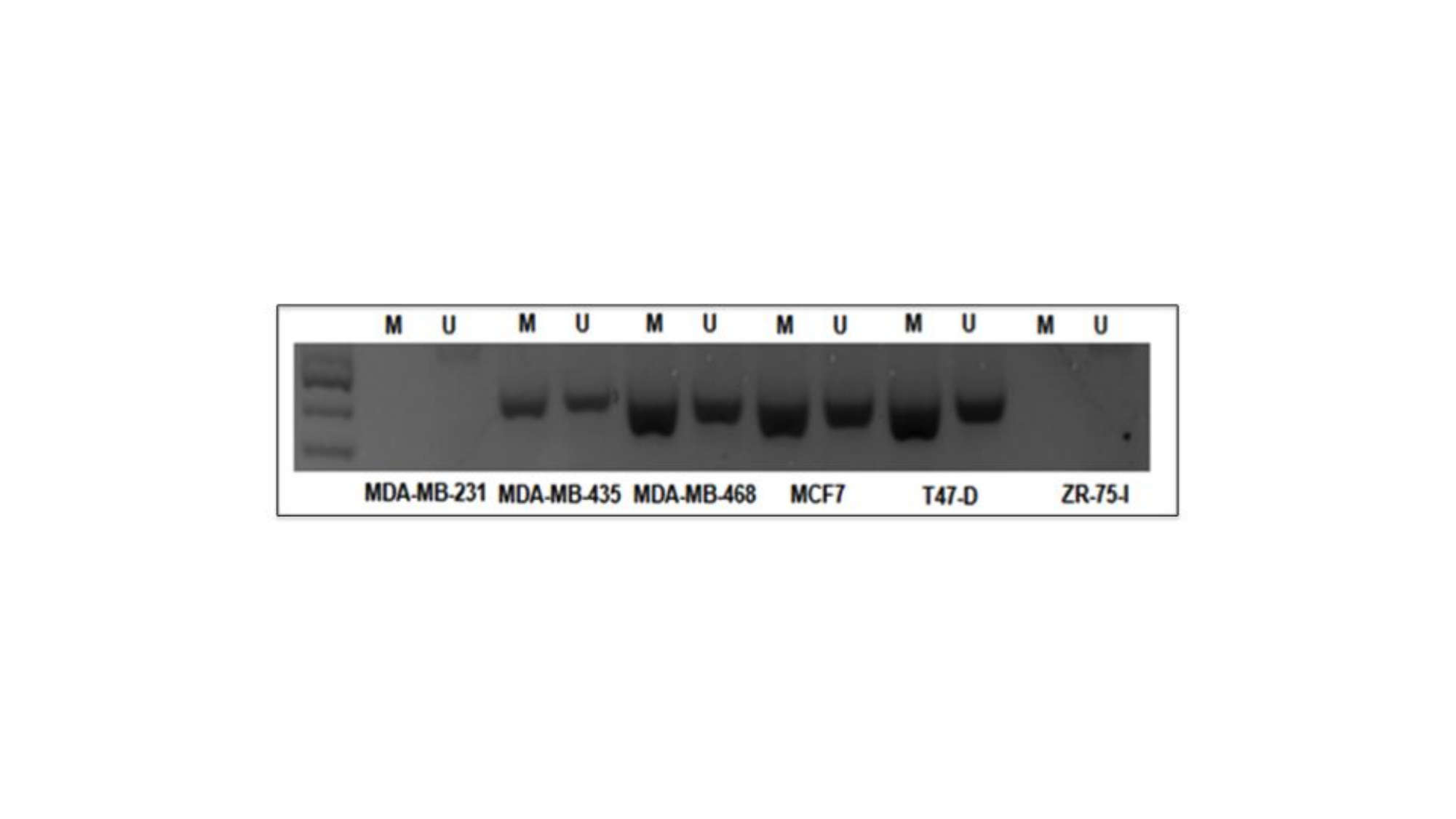

Supplement: S1 Fig — M, methylated; U, unmethymated. (PPTX) [file pone.0145647.s001.pptx]

## Slide 1
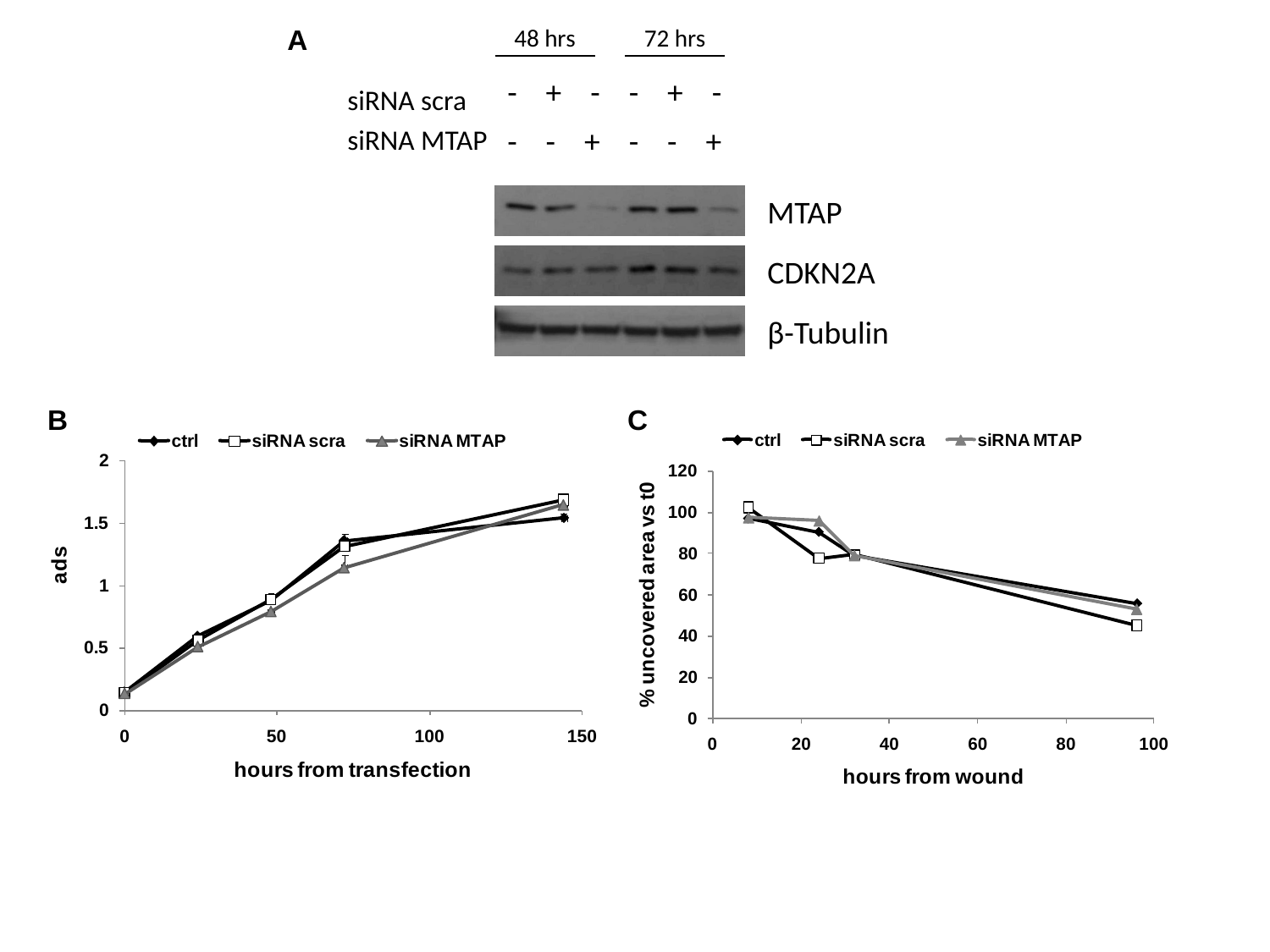

A
48 hrs
72 hrs
- + - - + -
siRNA scra
- - + - - +
siRNA MTAP
MTAP
CDKN2A
β-Tubulin
B
C

Supplement: S2 Fig — A. Western-blotting of MTAP, CDKN2A and β-Tubulin proteins at 48 and 72 hours from transfection with scramble siRNA and MTAP esiRNA. B. Proliferation of cells untransfected, transfected with scramble siRNA and transfected with MTAP esiRNA expressed as value of adsorbance at the wavelenght of 490 nm at different time points. C. Invasiveness of cells untransfected, transfected with scramble siRNA and transfected with MTAP esiRNA expressed as percentage of the uncovered area versus the initial one at different times from the wound. (PPTX) [file pone.0145647.s002.pptx]
