# Supplementary material for: Characterization of MTAP Gene Expression in Breast Cancer Patients and Cell Lines
Source: PLoS One. 2016 Jan 11;11(1):e0145647. doi: 10.1371/journal.pone.0145647 (PMC4709099; doi:10.1371/journal.pone.0145647)
Supplement: S1 Table — Primers sequences are in the sense 5’- 3’. m, methylated; u, unmethylated. (DOCX) [file pone.0145647.s003.docx]

| Gene | Forward | Reverse |
| --- | --- | --- |
| *RT-qPCR* |  |  |
| ACTB | GATGCAGAAGGAGATCACTGC | AGTACTTGCGCTCAGGAGGA |
| B2M | TGACTTTGTCACAGCCCAAGATA | CGGCATCTTCAAACCTCCA |
| MTAP | CCACCGCCGTGAAGATTGGAA | CCACCGCCGTGAAGATTGGAA |
| CDKN2A | CCACCGCCGTGAAGATTGGAA | AAGTTTCCCGAGGTTTCTCA |
| *MS-PCR* |  |  |
| MTAP m | TGTTTTTTAGGAATTAAGGGAAATAC | AACTACAAAATCTAACCCGACGAC |
| MTAP u | TTTTTAGGAATTAAGGGAAATATGT | CAACTACAAAATCTAACCCAACAAC |
